# Nutritional Outcomes of Bowel Lengthening Procedure in Patients with Short Bowel Syndrome

**DOI:** 10.3390/nu16101456

**Published:** 2024-05-12

**Authors:** Tena Niseteo, Mia Šalamon Janečić, Sara Sila, Anuka Torić, Laura Serdar, Stjepan Višnjić, Francisca Tolete Velcek, Marko Mesić, Iva Hojsak

**Affiliations:** 1 Referral Center for Pediatric Gastroenterology and Nutrition, Children’s Hospital Zagreb, 10000 Zagreb, Croatia; tniseteo@gmail.com (T.N.); miasalamon@hotmail.com (M.Š.J.); sara.sila0810@gmail.com (S.S.); 2 Pharmacy Unit, Children’s Hospital Zagreb, 10000 Zagreb, Croatia; anukapezo@gmail.com (A.T.); parenteralna@kdb.hr (L.S.); 3 Department of Surgery, Children’s Hospital Zagreb, 10000 Zagreb, Croatia; stjepanvisnjic@gmail.com (S.V.); mrkmesic@yahoo.com (M.M.); 4 Department of Surgery, School of Medicine, University of Zagreb, 10000 Zagreb, Croatia; 5 Division of Pediatric Surgery, State University of New York (SUNY) Downstate Health Sciences University, Brooklyn, NY 11203, USA; mdvelcek@gmail.com; 6 Department of Pediatrics, School of Medicine, University of Zagreb, 10000 Zagreb, Croatia; 7 Department of Pediatrics, School of Medicine, University J.J. Strossmayer, 31000 Osijek, Croatia

**Keywords:** intestinal failure, parenteral nutrition, serial transverse enteroplasty procedure

## Abstract

Background: Although parenteral nutrition (PN) significantly improves mortality rates in pediatric short bowel syndrome (SBS), long-term PN has many possible complications and impacts quality of life. Bowel lengthening procedures (BLPs) increase the contact surface of food and the intestinal mucosa and enable the better absorption of nutrients and liquids, possibly leading to a PN decrease. Methods: We retrospectively reviewed the data of patients with short bowel syndrome who underwent BLPs in the period from January 2016 to January 2022. Overall, eight patients, four male, five born prematurely, underwent BLPs. Results: There was a significant decrease in the percentage of total caloric intake provided via PN and PN volume after the BLPs. The more evident results were seen 6 months after the procedure and at the last follow-up, which was, on average, 31 months after the procedure. Two patients were weaned off PN after their BLPs. Patients remained well nourished during the follow-up. Conclusions: The BLP led to a significant decrease in PN needs and an increase in the food intake; however, significant changes happened more than 6 months after the procedure.

## 1. Introduction

Short bowel syndrome (SBS) is a condition marked by the bowel’s inability to digest and absorb nutrients adequately, leading to insufficient energy, nutrient, and fluid absorption [1]. Consequently, in children, this can hinder growth and development. The primary causes of SBS in pediatric patients typically involve conditions such as necrotizing enterocolitis (NEC), mid-gut volvulus (MGV), gastroschisis (GS), and intestinal atresia (IA), often resulting in a significant loss of intestinal tissue [2]. Several clinical features characterize SBS, such as malnutrition, dehydration, electrolyte abnormalities, and poor nutrient absorption [3]. Children with short bowel syndrome require parenteral nutrition (PN), along with enteral or oral food intake, to ensure proper growth and development. While PN notably reduces mortality rates in pediatric cases of short bowel syndrome, its long-term use can lead to various complications, such as potentially life-threatening sepsis and intestinal failure-associated liver disease (IFALD). Additionally, prolonged PN significantly affects quality of life [4].

The treatment of children with SBS is complex and requires a multidisciplinary team trained in intestinal rehabilitation. The primary goal of the treatment is to maximize enteral and/or oral intake by promoting intestinal adaptation and growth and reducing PN as much as possible while optimizing fluid and electrolyte balance. However, the intestinal adaptation and compensatory process often result in bowel dilatation, poorer motility, and stasis, often leading to vomiting and small intestinal bacterial overgrowth (SIBO). In many patients with congenital anomalies like intestinal atresia, the bowel is dilated from the very beginning, and problems with stasis and poor motility can happen earlier, causing poor bowel adaptation and an inability to increase enteral intake. If these complications develop and the treatment does not lead to better tolerance of enteral nutrition (EN) and food intake and, consequently, a reduction in PN, bowel lengthening procedures (BLPs) are indicated [5,6,7]. The main aim of surgery in SBS is to increase the mucosal surface area. The two most often used surgical procedures are the longitudinal intestinal lengthening and tailoring (LILT) technique described by Bianchi and serial transverse enteroplasty (STEP) [8]. Lengthening the bowel increases both the surface area of the intestinal mucosa and the duration of contact between food and the intestinal mucosa, thereby enhancing the absorption of nutrients and fluids. Simultaneously, reducing the bowel diameter decreases stasis and its potential negative effects. Collectively, these changes may facilitate a decrease in the need for parenteral nutrition (PN), thus supporting normal growth and development [9].

This retrospective study aimed to estimate the outcomes of bowel lengthening procedures in patients with SBS and their impact on PN.

## 2. Materials and Methods

The data of patients with short bowel syndrome who underwent BLPs using STEP or LILT methods in the period from January 2016 to January 2022 were retrospectively reviewed. All patients before the procedure used PN every day of the week. They received individualized PN bags compounded and distributed from the hospital pharmacy. Parenteral nutrition was defined according to the latest guidelines from The European Society for Pediatric Gastroenterology Hepatology and Nutrition (ESPGHAN) [10]. Data were assessed at five time points—2 months before BLP, at the time of BLP, 2 6 months after the BLP, and at the last follow-up. The following data were assessed: (1) anthropometric data, including body weight (BW), body height/length (BH/BL), BW for age Z-score, and BH/BL for age Z-score; (2) energy and fluid intake via parenteral, enteral, and oral routes; (3) bowel length pre- and postoperative procedure, which was determined by a surgeon during the operative procedure; (4) early complication occurrence (complications occurring within the first 3 months postoperation, mostly including bacteriemia, pseoudoileus/ileus, adhesions/strictures, high output, bleeding).

BW for age Z-score and BH/BL for age Z-score were assessed using World Health Organization (WHO) growth charts [11], and caloric intake was assessed using the Prodi Expert 5.6. dietetic software with a national and international database (Prodi, v. 5.7, Expert plus software, Nutri-Science, Stuttgart, Germany; 2011).

The primary outcome was to assess the decrease in energy and fluid intake via PN and the increase in EN and/or peroral food intake after the BLP. The secondary outcomes were the determination of early complication occurrence and bowel length gain.

*Statistics.* Differences in the variables were compared by paired tests, and due to non-normal distribution, Wilcoxon test was used. Data were analyzed using the SPSS 26.0 statistical program. *p* values below 0.05 were considered significant.

## 3. Results

Overall, eight patients, four male, five (63%) born prematurely, underwent BLPs. The cause of SBS was most commonly intestinal atresia (6/8 patients), which was accompanied by gastroschisis in 3 patients. One patient had mid-gut volvulus, and another one had extensive Hirschsprung disease affecting the whole colon and a part of the ileum. Indications for the lengthening procedure were the inability to increase the enteral nutrition/decrease parenteral nutrition intake and bowel dilatation that interfered with propulsive bowel motility. The mean age at BLP was 35.5 months (range 2 to 143 months), and 2 patients had their BLP at the age of below 6 months. Seven patients had STEP, and one had LILT BLP. The pre-BLP and post-BLP clinical patient data are shown in Table 1. The mean bowel length after the completion of the BLP was 47 cm (range 8–93 cm).

No central catheter-related bacteriemia occurred postoperatively, nor did it occur during the follow-up. All patients with IFALD (3/8) previous to the BLP had their liver disease resolved postoperatively.

Two months after the BLP, there was no significant difference in regard to weight or length for age Z score, PN volume, EN intake, and oral food intake. However, 6 months after the procedure, there was a significant decrease in the percentage of total caloric intake provided via PN (median 46 (range 30–60)% pre-BLP vs. 40 (range 27–61)% 6 months after BLP, *p* = 0.025).

**Figure 1:**
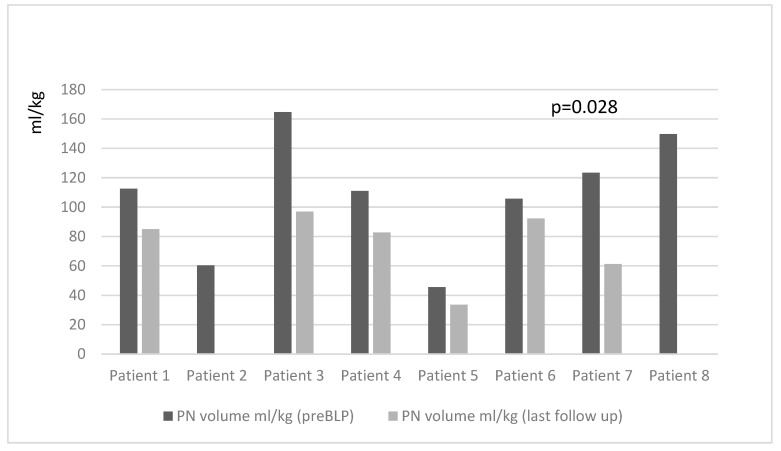
Changes in parenteral nutrition volume before and after (end of follow-up) bowel lengthening procedure by patient.

**Figure 2:**
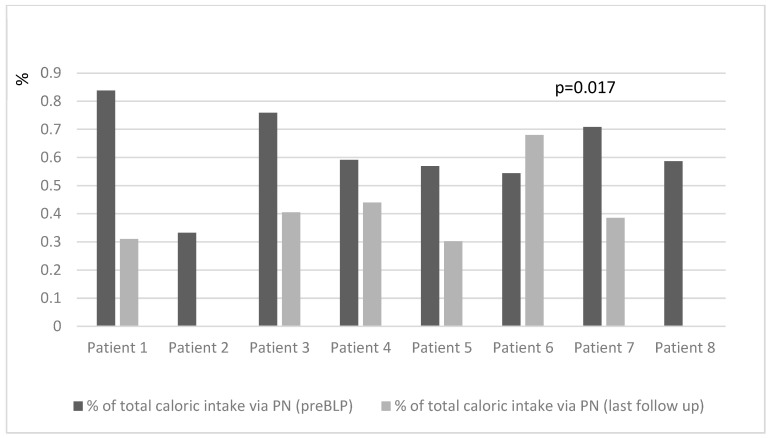
Changes in caloric intake via parenteral nutrition before and after (at the end of follow-up) bowel lengthening procedure by patient.

At the last follow-up (median 31 (range 9–60) months post-BLP), there was a significant decrease in the percentage of total caloric intake provided via PN (46 (30–60)% pre-BLP vs. 30 (0–44)% at the last follow-up, *p* = 0.017), PN volume mL/kg (85.6 (60–111) mL/kg pre-BLP vs. 33.6 (0–83) mL/kg last follow-up; *p* = 0.028), and PN energy kcal/kg (51.3 (36.8–65.7) kcal/kg pre-BLP vs. 19.2 (0–46) kcal/kg last follow-up; *p* = 0.017) (Figure 1 and Figure 2). Simultaneously, there was an increase in the peroral food energy intake (40 (31–49) kcal/kg pre-BLP vs. 44.2 (32–60) kcal/kg last follow-up; *p* = 0.017).

Two patients with the longest small bowel length after the BLPs were able to be weaned off PN. There was no significant change in the weight and length/height Z score during the follow-up.

The early complications in our patients were pseudoileus, hypoglycemia, oliguria, and high output, which were all resolved after symptomatic and conservative therapy. One patient developed chronic pancreatitis, not completely etiologically defined, that led to pancreatic insufficiency and the need for pancreatic enzyme replacement therapy. Two of our patients needed another surgery—one had another STEP procedure with adhesiolysis, and the second patient required adhesiolysis due to ileus and also had bowel tapering during that procedure. After the second surgery, both patients were fine, had no obstructive episodes, and had no problems associated with bowel motility.

## 4. Discussion

This is a single-center experience report on outcomes of the BLP in pediatric patients with SBS. This procedure resulted in several good outcomes. Our study showed that the increase in tolerance to food and EN was not significant in the first several months after the procedure, which is similar to the results described in other studies [12,13]. However, after 6 months, the BLP resulted in significantly increased EN and food tolerance. On the last follow-up visit in our study, which was, on average, after 31 months, PN’s contribution to total energy and fluid intake was significantly lower, which was in correspondence with other studies, regardless of the type of BLP [14]. Two out of our eight patients (25%) were weaned off PN, which is comparable to the study results of Barret et al. [12]; in both studies, the BLP was performed mostly using the STEP method. On the other hand, some studies have reported much better rates for weaning off PN, as there are some studies where 60 to 80% of patients reached enteral autonomy. This is probably due to the longer mean bowel length after the BLP, which was, in comparison to our study population (47 cm, range 8–93 cm), significantly longer (87 cm (31–270 cm) [13] to 103 cm (range, 24 to 375 cm)) [8]. A recent systematic review affirmed that bowel lengthening procedures (BLPs), regardless of the technique used (STEP or LILT), led to a reduction in parenteral nutrition (PN) dependency and an increase in enteral nutrition (EN) or oral food intake. This outcome was consistently reported across most studies, with PN being discontinued after a median duration of 180 days (ranging from 90 to 247.5 days) in 25% of patients. Furthermore, some studies indicated that after two or more years, 52% to 66% of patients with short bowel syndrome (SBS) were successfully weaned off PN [15].

Surgical bowel lengthening procedures increased the bowel length of our patients by, on average, 49%, but an increase of up to 85% was also found. This is in concordance with other studies where mostly STEP and LILT BLPs were analyzed and where a median increase in bowel length of 49.5–57% was reported [2,15].

In our study, all three patients with IFALD previous to the BLP had their liver disease resolved postoperatively. This was probably due to a significant decrease in PN, but it could have also been due to a reduction in SIBO and an increase in food intake and the tolerance of enteral nutrition. However, it is important to emphasize that the children in our study who underwent the BLP were not in a state of irreversible liver damage. Most other studies have reported IFALD recovery with the resolution of cholestasis after the BLP [9]. However, some studies have reported cases of deaths due to irreversible liver damage existing before the BLP [16].

Unlike in other reports, the change in the weight and length/height Z score during follow-up in our patients was not significant. Namely, in most other studies, a significant change in gained weight to a higher percentile, mostly after 12 or more months, was reported [8,9,12,13]. This could be explained by the fact that the majority of our patients before the BLP were not undernourished; moreover, they were well nourished and proportional due to the adequate provision of the needed energy, protein, vitamins, minerals, electrolytes, and fluids, mostly via PN, which enabled them to grow normally. After the BLP, except for two patients who reached enteral autonomy, the other patients received a combination of PN and EN and food in amounts adequate for enabling their growth at the same rate as when they were on higher PN intake.

We are aware of the several limitations of our study, which are mainly related to its small sample size and retrospective nature. However, data on patients with SBS treated with BLPs are scarce, and our study contributes by showing that even a smaller bowel elongation can result in better clinical outcomes. Furthermore, one could argue that adaptation of the bowel could happen at the end of follow-up as a consequence of bowel growth and adaptation without a BLP; however, the patients included in our study were patients with very short bowel lengths, and the majority of them were already over 12 months of age, meaning that they were in a “steady state” and that we could not decrease PN or patients that had complications due to dilated bowels. After surgery, their clinical status improved significantly, leading to the possibility of increased enteral intake. Therefore, in our opinion, performing a BLP on these selected patients was a crucial contributing factor leading to either enteral autonomy or its improvement.

## 5. Conclusions

In conclusion, the BLP led to a significant decrease in PN needs and an increase in food intake; however, significant changes happened more than 6 months after the procedure.

## Figures and Tables

**Table 1:** The pre-bowel lengthening procedure and post-bowel lengthening procedure clinical patient information.

	Patient 1	Patient 2	Patient 3	Patient 4	Patient 5	Patient 6	Patient 7	Patient 8
**Diagnosis**	IA	Hirschprung disease-whole colon and ileum	IA	MGV	IA, later MGV	GS + IA	GS + IA	GA + IA
**Colon (C)/ileocolonic valve (ICV)**	C+ ICV+	C− ICV−	C+ ICV+	C+ ICV+	C+ ICV−	C+ ICV−	C+ ICV−	C+ ICV−
**Number of abdominal operations before BLP**	1	6	4	2	6	1	4	3
**Pre-BLP BW/age Z score**	−1.54	−0.09	−4.05	−0.3	−0.89	−1.87	−2.98	−2.82
**Pre-BLP BH/age Z score**	/	1.16	/	0.42	−0.89	−0.32	−2.13	−3
**Age at BLP (months)**	2	56	10.5	24	138	3	37.5	13
**Bowel length pre-BLP (cm)**	6	65	26	15	25	25	43	35
**Bowel length post-BLP (cm) (bowel length gain%)**	8 (+33%)	93 (+43%)	33 (+27%)	21 (+40%)	37 (+48%)	31 (+24%)	62 (+44%)	65 (+85%)
**Early complications (first 3 months postoperatively)**	Pseudoileus—conservative therapy	0	Hypoglycemia, oliguria	0	Reoperation—2nd STEP, adhesiolysis (after 1st BLP)	2 reoperations due to ileus—adhesiolysis, tapering (after 1st BLP)	Chronic pancreatitis—pancreatic insufficiency	High output

BLP—bowel lengthening procedure, IA—intestinal atresia, GS—gastroschisis, MGV—midgut volvulus, and STEP—serial transverse enteroplasty.

## Data Availability

The original contributions presented in the study are included in the article, further inquiries can be directed to the corresponding author.
